# MOVEMENT AND HEALTH: NOVEL INSIGHTS INTO PHYSICAL ACTIVITY, ENERGY EXPENDITURE, AND MOTOR FUNCTION

**DOI:** 10.1093/geroni/igad104.1168

**Published:** 2023-12-21

**Authors:** Jennifer Schrack, Amal Wanigatunga, Nancy Glynn

## Abstract

Physical activity, energy expenditure, and motor function are well-established predictors of health and longevity with aging. Traditionally, most research using these measures has focused on speed of movement and intensity of activity. More recently, interest has grown in ways to refine these movement-related measures—and the methods used to analyze them—to improve predictive and discriminatory power for older adult health outcomes. This symposium will focus on measures derived from traditional in-lab assessments of motor function as well as free-living accelerometry, and their novel application across a variety of large U.S. cohort studies. Ms. Marino will present analyses on the links among heart rate variability, physical activity, and cognitive health in the Atherosclerosis Risk in Communities (ARIC) study. Dr. Dougherty will present the association of fine and gross motor function with MRI-derived brain volumes in the Baltimore Longitudinal Study of Aging (BLSA). Dr. Davoudi will present evidence on the link between physical activity quantities and variability with dementia in the National Health and Aging Trends Study (NHATS). Dr. Schumacher will compare measures of absolute and relative physical activity intensity with incident cardiovascular outcomes and all-cause mortality in the Objective Physical Activity and Cardiovascular Health (OPACH) Study. Finally, Dr. Wanigatunga will describe objectively measured physical activity quantities and patterns by self-reported fatigue levels and HIV status in the Multicenter AIDS Cohort Study (MACS). Collectively, these presentations will highlight novel ways to utilize motor function, energy expenditure, and physical activity data to advance prevention science and promote healthy aging. \ This is an Epidemiology of Aging Interest Group Sponsored Symposium.

